# Utilising Behavioural and Sensory Profiles and Associated Perinatal Factors to Identify Meaningful Subgroups in Autism Spectrum Disorder

**DOI:** 10.1007/s10803-024-06421-3

**Published:** 2024-06-06

**Authors:** Jane Shirley, James Rufus John, Alicia Montgomery, Andrew Whitehouse, Valsamma Eapen

**Affiliations:** 1 School of Clinical Medicine, University of New South Wales, Sydney, NSW Australia; 2 Ingham Institute of Applied Medical Research, Liverpool, NSW Australia; 3 Telethon Kids Institute, The University of Western Australia, Nedlands, WA Australia; 4 Academic Unit of Child Psychiatry, Liverpool Hospital, South Western Sydney Local Health District, Liverpool, NSW Australia

**Keywords:** Autism spectrum disorder, Paediatrics, Subgroups, Latent class analysis

## Abstract

The heterogeneity of autism spectrum disorder (ASD) clinically and aetiologically hinders intervention matching and prediction of outcomes. This study investigated if the behavioural, sensory, and perinatal factor profiles of autistic children could be used to identify distinct subgroups. Participants on the autism spectrum aged 2 to 17 years and their families were sourced via the Australian Autism Biobank (AAB). Latent class analysis was used to identify subgroups within this cohort, utilising twenty-six latent variables representing child’s behavioural and sensory features and perinatal factors. Four distinct subgroups within the sample (*n* = 1168) distinguished by sensory and behavioural autism traits and exposure to perinatal determinants were identified. Class 2 and Class 4, which displayed the greatest behavioural and sensory impairment respectively, were associated with the highest perinatal factor exposure. Class 1, labelled “Most behavioural concerns and moderate sensory and behavioural skills concerns” had mixed exposure to perinatal determinants while Class 3, named “Least sensory and behavioural skills concerns” had the least perinatal determinant exposure, indicating a directly proportional correlation between severity of clinical features and perinatal factor exposure. Additionally, association between specific exposures such as maternal mental illness in Class 1 and significant behavioural concerns was recognised. Identifying distinct subgroups among autistic children can lead to development of targeted interventions and supports. Close monitoring of children exposed to specific perinatal determinants for developmental differences could assist early intervention and supports.

## Introduction

Autism spectrum disorder (ASD) is a neurodevelopmental disorder (NDD) characterised by differences in social interaction and communication, intense and restrictive interests, and repetitive behaviours (Buescher et al., 2014; Leekam et al., 2011; Marotta et al., 2020). Autistic children typically present with differences in social reciprocity, aversion to change and unfamiliar environments, and social anxiety. This is often accompanied by speech disorders (e.g., language delay, echolalia, and lack of speech); limited ability to perform activities of daily living (e.g., learning, motor skills and memory); and other behavioural features (e.g., excitability and sensory hyper- or hyposensitivity) (Guthrie et al., 2013; Kim & Lord, 2013). Identifying and supporting these specific features early in life can augment a child’s ability to thrive.

A well-established characteristic of ASD is its heterogeneity in manifestations, severity, course, and therapeutic response of both diagnostic and associated psychiatric and behavioural presentations (Bryson et al., 2007; Zheng et al., 2020). Autistic children show clinical variability in behaviour, communicative functioning, rate of development, level of intellect, co-occurring conditions, and adaptive functioning (Masi et al., 2017; Zwaigenbaum et al., 2015). While heterogeneity is a recognised feature of the autism spectrum, its underlying aetiological mechanisms remain unclear (Uljarević et al., 2017; Vivanti et al., 2014). The proposed etiological pathway of ASD is not singular and involves a combination of genetic influence and environmental factors (Masi et al., 2017). The lack of clarity as to the underpinnings of the clinical diversity in autism has impeded the development of clinically effective treatments and supports (Uljarević et al., 2017; Vivanti et al., 2014). Alternatively, recognising distinct ASD subtypes could lead to streamlined education and therapy for specific needs and complexity levels (Charman, 2014). However, the usage of comprehensive sampling data gathered from multiple behavioural and etiological domains to identify overall subgroups in ASD is scarce.

This study aimed to identify meaningful subgroups in autistic children by assessing their behavioural, sensory, and perinatal factor profiles using the Australian Autism Biobank (AAB) - the largest Australian national data repository of detailed biological and clinical information about children on the autism spectrum, created to further autism discovery and research (Alvares et al., 2018). Our research questions were: (1) Can children with ASD be subtyped based on their behavioural, sensory and perinatal factor profiles? and (2) Comparing the subtypes, are there differences in clinical profile vis-à-vis exposure to perinatal factors? It is expected that identifying homogenous subgroups with similar clinical profile and exposure to perinatal determinants will ultimately help to bring targeted interventions and supports for distinct subgroups of autistic children, specifically tailored to the unique profile of each subgroup. Further, finding precision medicine approaches for autistic children may result in improved course, treatment outcomes and quality of life in adulthood (Loth et al., 2017; Woolfenden et al., 2022).

## Methods

### Study Design and Participants

This study is a secondary data analysis of the AAB, a collection of phenotypic and biological data of children on the autism spectrum (aged 2–17 years) along with their siblings, parents, and unrelated controls without autism (Alvares et al., 2018). Participants were recruited between 2013 and 2018 across four states of Australia: Telethon Kids Institute, Perth, Western Australia; Olga Tennison Autism Research Centre, La Trobe University, Victoria; University of New South Wales, Sydney, NSW; and Lady Cilento Children’s Hospital, Brisbane, Queensland (Alvares et al., 2018). All children with an ASD diagnosis and when feasible, family members were invited to participate in the AAB. Clinical data and biological samples were collected at clinical facilities in each site or by completed assessments and samples sent by mail for remote families (Alvares et al., 2018). For the purpose of this study, our analysis pertained to information obtained from detailed history, clinical and behavioural data of two participant groups, (1) ASD probands: Children with a clinically confirmed diagnosis of ASD, and (2) ASD queries: Children suspected to have ASD but did not meet the Diagnostic and statistical manual of mental disorders-5® criteria (American Psychiatric Association, 2013). Two participant groups in the AAB, namely siblings of ASD probands and controls were not included in this analysis. No exclusion criteria were applied with regard to thoroughness of materials submitted.

### Data Collection

Clinical and behavioural data within the AAB was collected at entry, via standardised clinical assessments appraising each participant’s clinical features, cognitive functioning, and physical development. To achieve the aim of this study, the analysis was limited to data associated with only the behavioural, sensory, and perinatal profiles of the children. This was achieved via multiple questionnaires completed by each participant’s parents or caregivers, namely the Vineland Adaptive Behaviour Scale-II (VABS-II) measuring adaptive functioning and behavioural manifestations, Short Sensory Profile-2 (SSP-2) for sensory processing abnormalities, and a custom family history questionnaire (FHQ) for sociodemographic data and detailed past child and family histories (Dunn, 2014; Sparrow et al., 2005). The data pertaining each participant included in the primary analysis was entered only once.

### Latent Variables

The primary analysis of this study was a latent class analysis (see Statistical analyses section), which utilises the child’s age and twenty-six latent variables representing the behavioural (VABS-II), sensory (SSP-2), and perinatal (FHQ) factorial domains to create meaningful autism subgroups using the AAB data (Dunn, 2014; Sparrow et al., 2005).

#### Behavioural Profiles

Five composite-based scores generated by the VABS-II questionnaire were used to represent five behavioural domains: communication, socialisation, daily living, motor skills, and maladaptive behaviours (including temper tantrums, aggression, or disobedience). The scores of each domain are based on a five-level scale: low, moderately low, adequate, moderately high and high. The communication category measures receptive, expressive and written communication skills while the socialisation domain covers interpersonal relationships and play or leisure time. The daily living skills domain represents domestic and community interaction skills and the motor skills level measures gross and fine motor abilities.

#### Sensory Profiles

Four composite-based quadrants of seeking/seeker, avoiding/avoider, sensitivity/sensor, and registration/bystander generated by the SSP-2 questionnaire were employed to reflect sensory profiles of participants. The scoring system of each quadrant has five tiers: much less than others, less than others, just like the majority of others, more than others and much more than others. Seeking/seeker tendencies involve children seeking additional sensory input (e.g., touching objects in their environment or additional movement) throughout the day whereas the avoiding/avoider group are overwhelmed by and avoid sensory input, which is expressed through frustration precipitated by change and avoidance of large gatherings. High scores in the sensitivity/sensor classification suggest a higher rate of sensory detection and thus hyper-reactivity, manifesting as avoiding being messy and loud settings. Registration/bystander tendencies are results of missing or reduced detection of sensory input and include needing instructions repeated to them multiple times or frequent bumps into obstacles. The classifications are four distinct measures of sensory hyper-reactivity, hypo-reactivity, and seeking behaviour, which are traits significantly prevalent among autistic children (Ben-Sasson et al., 2009; Lane et al., 2014).

#### Perinatal Factors

Seventeen latent variables were chosen to represent the perinatal factors, segregated as antenatal, natal, and postnatal factors. These variables were selected following assessment of existing literature for significant and relevant perinatal determinants (Bilder et al., 2009; Yong et al., 2021).

The selected antenatal factors were mother’s age at birth (median age < 34 years or ≥ 34 years), mother’s body mass index (BMI) [BMI under 18.5 (Underweight), 18.5 to 24.9 (Healthy), 25.0 to 29.9 (Overweight) or 30.0 or higher (Obese)], raised blood pressure (BP) during pregnancy (yes/no), mother’s smoking history in the year before pregnancy (never, 1 to 5 daily or > 5 daily), mother’s prescription status during pregnancy (yes/no) and maternal history of physical and mental health illnesses. Physical health conditions were segregated into immune or inflammatory conditions (e.g., systemic lupus erythematosus, hepatitis, rheumatic fever) (yes/no) and non-communicable diseases (e.g., asthma, diabetes, heart disease, hypertension) (yes/no). Maternal mental illnesses (yes/no) investigated in the family history questionnaire included but were not limited to anxiety, depression, and schizophrenia.

Natal variables selected included birth factors while mothers were pregnant with the participating child. These included induction of labour (yes/no), type of delivery (caesarean or vaginal), length of term (preterm, early term, full term or late term), vaginal spotting or bleeding (yes/no) and complications during pregnancy (yes/no).

The predisposing postnatal variables selected were problems in child’s first week of life (e.g., jaundice, feeding problems, infections) (yes/no), unusual development during child’s first six months of life (e.g., maladaptive behaviours, feeding issues, behavioural abnormalities) (yes/no), postnatal conditions (yes/no) and baby’s birthweight (low or normal to high birthweight). Postnatal conditions that were investigated in the FHQ included but were not limited to intellectual disability, global developmental delay, cerebral palsy, and encephalitis.

### Statistical Analyses

Latent class analysis (LCA) is defined as an empirical approach to identifying underlying subtypes or subgroups within patterns of data consisting of latent variables coded categorically (Weller et al., 2020). The analysis involved creating various latent class fit-models, starting with a one-class model and then creating subsequent models by increasing the number of classes. Finally, the model that best illustrated the latent structure of the data was selected. This was accomplished by appraising the specific goodness of fit statistics and interpretability of all the derived latent class models. The statistics included the log-likelihood ratio (LLR), where a higher value is favoured, Bayesian Information Criterion (BIC) and Akaike Information Criterion (AIC), of which smaller values indicate a better fit and parsimony (Weller et al., 2020). The Lo-Mendell-Rubin Likelihood Ratio Test (LMR-LRT) provides a p value, which if statistically significant, indicates that the model better represents the latent structure of data than the model with one fewer class (Nylund et al., 2007). The entropy is a value between 0 and 1, where a greater value indicates more precise classification of individuals into subgroups (Petersen et al., 2019). LCA uses the actual probability of each score in the categorical variables to assign individuals to their most likely class. The differing probabilities of the variables were compared between classes to find distinct and meaningful subgroups. Statistical analysis was executed with both JMP Pro 17 (“JMP® Version 17,” 1989–2023) and RStudio (RStudio 2023.03.1 + 446).

## Results

This study used a sample of 1168 participants, including ASD-probands and ASD-queries. The mean (SD) age of the sample was 7.6 (3.9) years and had a higher proportion of males (67.8%). The demographics of the study population are outlined in Table 1. The respective probabilities for each score under each latent variable are available in the Supplementary Table 1.

**Table 1:** Demographics of the Australian autism biobank cohort

	ASD-Probands and ASD-Queries	
	*N* = 1168	%
Child’s age in years		
* Mean (SD)*	7.6 (3.9)	
Child’s Sex		
* Male*	792	67.8%
* Female*	226	19.3%
* Missing*	150	12.8%
Main language spoken		
* English*	951	81.4%
* Others*	65	5.6%
* Missing*	152	13.0%
Maternal ethnicity		
* Caucasian*	762	65.2%
* Aboriginal*	7	0.6%
* Others*	179	15.3%
* Missing*	220	18.8%
Mother’s education level		
* Up to school level education*	245	21.0%
* Trade or technical certification*	171	14.6%
* University degree*	438	37.5%
* Missing*	314	26.9%
Paternal ethnicity		
* Caucasian*	770	65.9%
* Aboriginal*	0	0.8%
* Others*	160	13.7%
* Missing*	229	19.6%
Father’s education level		
* Up to school level education*	282	24.1%
* Trade or technical certification*	244	20.9%
* University degree*	341	29.2%
* Missing*	301	25.8%
Total family income		
* ≤$40,000*	103	8.8%
* >$40,000*	726	62.2%
* Missing*	339	29.0%

### Latent Class Analysis

**Fig. 1:**
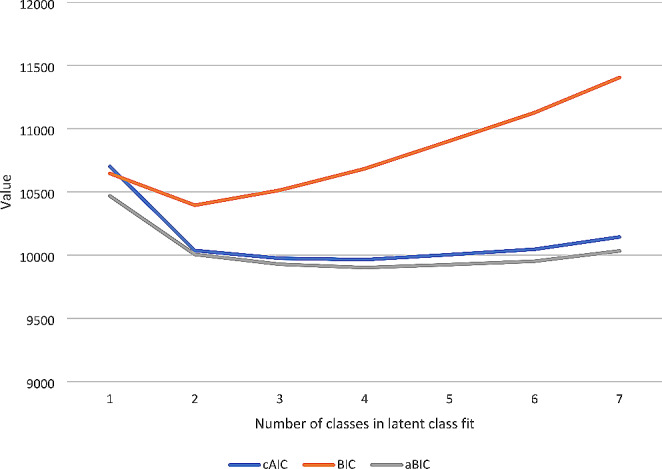
Line chart of goodness of fit statistics of latent class models of autism-probands and autism-queries in the Australian Autism Biobank

Table 2 outlines the goodness of fit statistics used to find the best-fitting latent class model. After comparison of the latent class models generated by LCA of the 26 latent variables, the four-class model was deemed the most appropriate to describe the identified subgroups. The LMR-LRT suggested that the four-class model was not a statistically better fit than the five-class model (*p* < 0.001) (Table 2). However, the gradual decrease of the adjusted BIC (aBIC) and consistent AIC (cAIC) values with each addition of class till the four-class model and the four-class model having a lower BIC value than the five-class model (Fig. 1), indicated that the four-class model was the best fit to explain the latent class structure of the sample. Further, the four-class model also had a high entropy value of 0.892, reflecting good separation between the classes.

The subgroups created by the four-class model suggested an association between the perinatal factors and the behavioural and sensory profiles in each group, explaining the distinct clinical phenotype of each subgroup. Identified subgroups were labelled Class 1: “Most behavioural concerns with moderate sensory and adaptive behaviour skills concerns”, with mixed exposure to perinatal determinants and in particular high rates of maternal mental illness; Class 2: “Most behavioural skills concerns”, with significant exposure to natal and postnatal determinants; Class 3: “Least sensory and behavioural skills concerns”, with the least exposure to perinatal determinants and Class 4: “Most sensory impairment”, with significant exposure to perinatal determinants. The probabilities of all the participants in each class are listed in Table 3 and the main variations that distinguish the classes are described in Table 4.

**Table 2:** Latent class fit statistics

Classes	Log-likelihood	cAIC^a^	BIC^b^	aBIC^c^	LMR-LRT^d^ (*p*)	Entropy
1	-5170.260	10701.538	10645.54	10468.047	-	-
2	-4889.555	10036.441	10394.59	10005.110	< 0.001	0.916
3	-4793.759	9974.653	10513.46	9927.517	< 0.001	0.83
4	-4723.597	9964.134	10683.60	9901.194	< 0.001	0.892
5	-4678.405	10003.554	10903.68	9924.810	< 0.001	0.914
6	-4634.849	10046.245	11127.03	9951.697	< 0.001	0.907
7	-4618.549	10143.451	11404.90	10033.099	< 0.001	0.828

^a^Consistent Akaike Information Criterion

^b^Bayesian Information Criterion

^c^Adjusted Bayesian Information Criterion (BIC)

^d^Lo-Mendell-Rubin Likelihood Ratio Test

**Table 3:** Variable probabilities by latent class for children with diagnosed or suspected autism in the Australian Autism Biobank – four-class model

	Class one	Class two	Class three	Class four
	(28.45%)	(33.19%)	(29.74%)	(8.62%)
Child’s age				
0 to 5 years	0.5016	0.6532	0.7782	0.5421
6 to 12 years	0.4811	0.2714	0.1928	0.4579
13 to 17 years	0.0173	0.0754	0.0291	0
**Behavioural profiles**				
Communication Adaptive Level				
1 low	0.0917	0.6679	0.0602	0.4507
2 moderately low	0.5472	0.3321	0.3116	0.3951
3 adequate	0.3612	0	0.5556	0.1542
4 moderately high	0	0	0.0726	0
5 high	0	0	0	0
Socialisation Adaptive level				
1 low	0.3135	0.673	0.098	0.5234
2 moderately low	0.4819	0.318	0.3379	0.424
3 adequate	0.2046	0.009	0.4914	0.0526
4 moderately high	0	0	0.0726	0
5 high	0	0	0	0
Motor Adaptive Level				
1 low	0.1211	0.4612	0.0532	0.4829
2 moderately low	0.3903	0.4319	0.3281	0.4155
3 adequate	0.4886	0.1069	0.5315	0.0508
4 moderately high	0	0	0.0726	0.0508
5 high	0	0	0.0145	0
Daily Living Adaptive level				
1 low	0.1296	0.6966	0	0.6756
2 moderately low	0.653	0.2903	0.4092	0.1702
3 adequate	0.2023	0.0131	0.4599	0.1542
4 moderately high	0.0152	0	0.0872	0
5 high	0	0	0.0436	0
Maladaptive level				
1 clinically significant	0.7309	0.4098	0.079	0.1
2 elevated	0.2398	0.5235	0.5886	0
3 average	0.0294	0.0667	0.3324	0
**Sensory profiles**				
Seeking/Seeker Classification				
1 much less than others	0	0	0	0
2 less than others	0	0	0	0
3 just like the majority of others	0.1043	0.3446	0.7603	0.0508
4 more than others	0.2474	0.3633	0.2086	0.1591
5 much more than others	0.6483	0.2921	0.0312	0.79
Avoiding/Avoider Classification				
1 much less than others	0	0	0	0
2 less than others	0	0	0	0
3 just like the majority of others	0.0172	0.1815	0.5339	0
4 more than others	0.0577	0.4216	0.3837	0
5 much more than others	0.9251	0.3969	0.0825	1
Sensitivity/Sensor Classification				
1 much less than others	0	0	0	0
2 less than others	0	0	0	0
3 just like the majority of others	0.0138	0.1264	0.5994	0
4 more than others	0.0976	0.344	0.3315	0.1016
5 much more than others	0.8886	0.5295	0.0691	0.8984
Registration/Bystander Classification				
1 much less than others	0	0	0	0
2 less than others	0	0	0.0581	0
3 just like the majority of others	0.0386	0.3758	0.7428	0.1066
4 more than others	0.2364	0.2861	0.1484	0
5 much more than others	0.7249	0.3382	0.0507	0.8934
**Perinatal factors**				
***Antenatal factors***				
Mother’s age at birth				
1 < 34	0.6719	0.5983	0.5869	0.758
2 ≥ 34	0.3281	0.4017	0.4131	0.242
Mother’s BMI				
1 Under 18.5 (Underweight)	0.0305	0	0.029	0
2 18.5–24.9 (Healthy)	0.3691	0.3513	0.5209	0.2827
3 25.0–29.9 (Overweight)	0.3037	0.3324	0.2338	0.0555
4 30.0 or higher (Obese)	0.2967	0.3163	0.2162	0.6618
Immune or inflammatory conditions in mother				
1 No	0.8387	0.7991	0.8903	0.7345
2 Yes	0.1613	0.2009	0.1097	0.2655
Non-communicable diseases in mother				
1 No	0.5509	0.6709	0.7356	0.6088
2 Yes	0.4491	0.3291	0.2644	0.3912
Mental health conditions in mother				
1 No	0.4058	0.6584	0.7938	0.2784
2 Yes	0.5942	0.3416	0.2062	0.7216
Mother’s smoking history in the year before pregnancy				
1 Never	0.8266	0.8683	0.8599	0.6583
2 1–5 daily	0.0723	0.0789	0.0649	0.1342
3 > 5 daily	0.1011	0.0528	0.0752	0.2075
Raised blood pressure during pregnancy				
1 No	0.919	0.8181	0.8879	0.603
2 Yes	0.081	0.1819	0.1121	0.397
Mother’s prescription status during pregnancy				
1 No	0.5855	0.6043	0.5637	0.4435
2 Yes	0.4145	0.3957	0.4363	0.5565
***Natal factors***				
Induction of labour				
1 No	0.5477	0.5245	0.6718	0.7101
2 Yes	0.4523	0.4755	0.3282	0.2899
Type of delivery				
1 Vaginal	0.7377	0.467	0.5608	0.2982
2 Caesarean	0.2663	0.533	0.4392	0.7018
Length of term				
1 Preterm	0.0167	0.154	0.0722	0.6078
2 Early term	0.2031	0.2736	0.3313	0.2321
3 Full term	0.5409	0.4378	0.4395	0.1601
4 Late term	0.2394	0.1345	0.157	0
Vaginal spotting or bleeding				
1 No	0.6996	0.655	0.718	0.4403
2 Yes	0.3004	0.345	0.282	0.5597
Complications during pregnancy				
1 No	0.6483	0.5467	0.5648	0
2 Yes	0.3517	0.4533	0.4352	1
***Postnatal factors***				
Problems in first week of life				
1 No	0.6747	0.6985	0.7052	0.1926
2 Yes	0.3253	0.3015	0.2948	0.8074
Unusual development during child’s first six months of life				
1 No	0.4788	0.4773	0.7387	0.3335
2 Yes	0.5212	0.5227	0.2613	0.6665
Postnatal conditions				
1 No	0.5317	0.3388	0.6797	0.352
2 Yes	0.4683	0.6612	0.3203	0.648
Baby birthweight				
1 Low birthweight	0	0.0394	0.0728	0.5048
2 Normal – High birthweight	1	0.9606	0.9272	0.4952

**Table 4:** Summary of subtype variations for children with diagnosed or suspected autism in the Australian Autism Biobank, based on the four-class latent model

	Class one: “Most behavioural concerns with moderate sensory and adaptive behaviour skills concerns” with mixed exposure to perinatal determinants	Class two: “Most behavioural skills concerns” with significant exposure to natal and postnatal determinants	Class three: “Least sensorily and behavioural skills concerns”, with least exposure to perinatal determinants.	Class four: “Most sensorily impairment” with significant exposure to perinatal determinants.
Social Communication levels	**Similar** probability of low to moderately low communication and socialisation compared to the overall group.	**Highest** probability of low communication and socialisation adaptive levels.	**Lowest** probability of low to moderately low communication and socialisation adaptive levels.	**Higher** probability of low to moderately low communication and socialisation than overall group.
Adaptive functioning	**Similar** probability of low to moderately low daily and motor adaptive levels compared to overall group.	**Highest** probability of low to moderately low adaptive functioning.	**Lowest** probability of low to moderately low daily and motor adaptive functioning.	**Higher** probability of low to moderately low adaptive functioning than overall group.
Maladaptive behaviour	**Highest** probability of clinically significant maladaptive behaviour levels.	**Higher** probability of clinically significant maladaptive behaviour scores than the overall group.	**Lower** probability of clinically significant and elevated maladaptive behaviour than the overall group.	**Lowest** probability of clinically significant and elevated maladaptive behaviours.
Seeking/seeker classification	**Higher** probability of having much more seeking/seeker tendencies than the overall group.	Probability of having more or much more seeking/seeker tendencies than a behaviourally typical person is **similar** to overall group.	**Lowest** probability of having more to much more seeking/seeker tendencies compared to a behaviourally typical person.	**Highest** probability of having much more seeking/seeker tendencies compared to others.
Avoiding/Avoider Classification	**Higher** probability of having much more avoiding/avoider tendencies than the overall group.	Probability of having much more avoiding/avoider tendencies compared to a behaviourally typical person is **similar** to the overall group.	**Lowest** probability of having much more avoiding/avoider tendencies compared to a behaviourally typical person.	**Highest** probability of having much more avoiding/avoider tendencies compared to a behaviourally typical person.
Sensitivity/Sensor Classification	**Higher** probability of scoring to have much more sensitivity/sensor tendencies than the overall group.	Probability of having much more sensitivity/sensor tendencies compared to a behaviourally typical person is **similar** to the overall group.	**Lowest** probability of having much more sensitivity/sensor tendencies compared to a behaviourally typical person.	**Highest** probability of having much more sensitivity/sensor tendencies compared to a behaviourally typical person.
Registration/Bystander Classification	**Highest** probability of having registration/bystander tendencies.	Probability of having much more registration/bystander tendencies compared to a behaviourally typical person is **similar** to the overall group.	**Lowest** probability of having much more registration/bystander tendencies compared to a behaviourally typical person.	Probability of having much more registration/bystander tendencies compared to a behaviourally typical person is **higher** than the overall group.
Mother’s BMI	**Similar** probability of mother being overweight or obese compared to the overall group.	Probabilities of mother’s BMI being obese or overweight is **similar** to the overall group.	Probability of mother’s BMI being obese or overweight is the **lowest** in the overall group.	**Highest** likelihood of mother’s BMI being obese or overweight than the overall group.
Immune or inflammatory conditions in mother	**Similar** likelihood of mother having an immune or inflammatory condition compared to overall group.	**Higher** probability of maternal immune or inflammatory illness than the overall group.	**Lowest** likelihood of mother having an immune or inflammatory condition.	**Highest** probability of presence of immune or inflammatory illness in mother compared to overall group.
Presence of non-communicable diseases in mother	**Highest** likelihood of mother having a non-communicable disease.	**Similar** probability of presence of non-communicable diseases in mother compared to overall group.	**Lowest** probability of mother having a non-communicable disease.	Probability of maternal non-communicable diseases is **higher** to overall group.
Mental health of mother	**Higher** probability of mother having mental health illnesses than the overall group.	**Lower** probability of mother having a mental health illness than the overall group.	**Lowest** probability of mother having mental illnesses.	**Highest** probability of mother having a mental health illness.
Raised blood pressure during pregnancy	**Lowest** probability of mother having raised blood pressure during pregnancy.	Probability of mother’ blood pressure being raised during pregnancy is **similar** to overall group.	**Lower** probability of mother having a raised blood pressure during pregnancy than overall group.	**Greatest** probability of mother having a raised blood pressure during pregnancy.
Induction of labour	**Higher** likelihood of induction of labour compared to overall group.	**Highest** probability of mother’s labour being induced compared to overall group.	Probability of induction of labour is **similar** compared to the overall group.	**Lower** probability of induction of labour compared to the overall group.
Caesarean delivery	**Lower** probability of caesarean delivery compared to the overall group.	**Higher** probability of caesarean delivery compared to the overall group.	Probability of caesarean delivery is **similar** to the overall group.	**Highest** probability of caesarean delivery compared to the overall group.
Vaginal spotting or bleeding	**Similar** probability of mother having natal vaginal spotting or bleeding compared to the overall group.	**Higher** probability of mother having natal vaginal spotting or bleeding than the overall group.	**Lowest** probability of mother having natal vaginal spotting or bleeding.	**Highest** probability of mother having natal vaginal spotting or bleeding.
Complications during pregnancy	**Similar** probability of mother having complications during pregnancy compared to overall group.	**Higher** probability of mother having complications during pregnancy compared to overall group.	**Similar** probability of mother having complications during pregnancy compared to overall group.	**Highest** probability of mother having complications during pregnancy than overall group.
Developmental problems during initial period of life	Probability of unusual development during first 6 months of life is **higher** and that of problems in first week of life is **lower** than the overall group.	Probability of unusual development during first 6 months of life is **higher** and that of problems in first week of life is **lower** than the overall group.	Probability of development concerns during first 6 months of life and problems in first week of life is the **lowest** in the overall group.	**Highest** probability of problems in the first week of life and problems in first 6 months of life.
Postnatal conditions	Probability of developing postnatal conditions is **similar** compared to the overall group.	**Highest** probability of developing postnatal conditions compared to the overall group.	Probability of developing postnatal conditions is the **lowest** in the overall group.	Probability of developing postnatal conditions is **higher** than the overall group.

Subtype differences in variables (compared to overall group) are highlighted in bold

### Class Descriptions

In this study, Class 1 (28.45%) was labelled “Most behavioural concerns with moderate sensory and adaptive behaviour skills concerns”, which showed mixed exposure to perinatal determinants and particularly high probability of maternal mental illness. Compared to the overall group, Class 1 members scored “low” or “moderately low” across the behavioural adaptive skills items of the VABS-II questionnaire. However, Class 1 had the highest probability of 0.7309 of exhibiting clinically significant or elevated maladaptive behaviours compared to overall group. Children in Class 1 were more likely to score “much more than others” in the seeking/seeker, avoiding/avoider, sensitivity/sensor and most likely to score “much more than others” in the registration/bystander classifications of the SSP-2, with probabilities of 0.6483 for seeking/seeker, 0.9251 for avoiding/avoider, 0.8886 for sensitivity/sensor, and 0.7249 for registration/bystander. This was coupled with a mixed exposure to perinatal factors. Relative to the overall group, mothers of individuals in Class 1 were more likely to report a history of non-communicable diseases, mental health illnesses, and an induced labour with probabilities of 0.4491, 0.5942 and 0.4523 respectively. They were also less likely to have experienced raised blood pressure during pregnancy and a caesarean delivery with probabilities 0.0810 and 0.2663 respectively. Class 1 had a higher probability of unusual development during the first six months of life with a probability of 0.5212 but a lower probability of problems in the first week of life with a value of 0.3253 compared to the overall group.

Class 2 (33.19%) was characterised as having “Most behavioural skills concerns”, with significant exposure to natal and postnatal determinants. This subgroup had the highest likelihood of scoring ‘low’ or ‘moderately low’ in the communication, socialisation, daily living, and motor adaptive categories of the VABS-II questionnaire, with the mean probability being 0.9678 across all adaptive functioning items. Class 2 also presented with a higher probability of clinically significant maladaptive tendencies than the overall group, with a value of 0.4098. While the probabilities of exposure to majority of antenatal factors were either similar or lower compared to the overall group, Class 2 had a higher likelihood of exposure to postnatal determinants, such as developmental concerns during child’s first six months of life and postnatal conditions, with probabilities of 0.5227 and 0.6612 respectively. With respect to natal factors, mothers of this subgroup were most likely to have had an induced labour with a probability of 0.4755 and more likely to have had a caesarean delivery, vaginal spotting or bleeding and complications during pregnancy than the overall group, with probabilities of 0.5330, 0.3450, and 0.4533 respectively.

Class 3 (29.74%), was labelled as “Least sensory and behavioural skills concerns” and reported least exposure to perinatal determinants. Class 3 presented with the lowest likelihood and mean probability of 0.05838 to score “much more than others” across all sensory classifications of the SSP-2. This was paired with lowest probabilities of scoring ‘low’ or ‘moderately low’ in the behavioural adaptive items with 0.3718 for communication, 0.4359 for socialisation, 0.4092 for daily living, and 0.3813 for motor skills. Class 3 was also less probable than the overall group to have ‘clinically significant’ or ‘elevated’ maladaptive behaviours, with a probability of 0.6676. Class 3 presents with a collectively lower probability of exposure to maternal antenatal factors than the overall group, with the probabilities being 0.4500 for maternal history of being overweight or obese, 0.1097 for immune or inflammatory conditions, 0.2644 for non-communicable diseases, 0.2062 for mental health illnesses, and 0.1121 for raised blood pressure during pregnancy. Mothers of children in Class 3 additionally had the lowest probability of natal vaginal spotting or bleeding. This was accompanied by the smallest probabilities of exposure to postnatal factors, with 0.2948 for problems in first week of child’s life, 0.2613 for unusual development in the first six months of life and 0.3203 for postnatal conditions.

Class 4 (8.62%), was identified as “Most sensory impairment”, with significant exposure to perinatal determinants. Class 4 had the highest percentage of individuals scoring “much more than others” across the seeking/seeker, avoiding/avoider and sensitivity sensor, classifications of the SSP-2, ranging from 79.0 to 100%. Participants in this subgroup were also more likely to have lower scores in the behavioural adaptive domains including socialisation, communication, daily living, and motor skills compared to the overall group. This was coupled with the greatest likelihood of exposure to antenatal factors, such as maternal obesity and hypertension during pregnancy, with probabilities of 0.6618 and 0.3970 respectively. Participants in this subgroup had the highest probability of a maternal history of immune or inflammatory conditions and mental illness and a probability higher than the overall group for non-communicable diseases with values 0.2655, 0.7217 and 0.3912 respectively. This was associated with probabilities of natal and postnatal variables either higher than or highest in the overall group, with 0.7018 for caesarean delivery, 1.000 for complications during pregnancy, 0.5597 for vaginal spotting or bleeding, 0.8.74 for problems in first week of child’s life, 0.6665 for unusual development during first six months of life, and 0.6480 for postnatal conditions.

## Discussion

This study presents latent classes of children with autism differentiated by their behavioural, sensory, and perinatal profiles and the patterns between them. In doing so, we identified four distinct subgroups of autistic children. Class 1 presented with variable probabilities which were neither in the highest nor lowest categories, justifying its classification as moderate sensory and behavioural concerns with mixed exposure to perinatal determinants. Children in Class 2 were found to have the most adaptive behavioural skills concerns, with significant exposure to natal and postnatal determinants. Class 3 showed the least sensory and behavioural skills concerns with the lowest probability of exposure to perinatal determinants while Class 4 displayed the highest level of sensory impairment with the highest probability of exposure to most perinatal determinants. Each class showed a distinct behavioural and/or sensory profile directly corresponding to exposure to specific determinants in the antenatal, natal, and postnatal period.

Currently available studies are significantly diverse in terms of sample size, statistical methods, and the number and type of latent variables used to describe latent structure, making comparable existing literature scarce. Class 1 showed a gradient of symptom severity across the different variables, with classes being least to most behaviourally and/or sensorily affected. Despite multiple studies identifying subgroups predominantly differentiated by autism symptom severity (Cholemkery et al., 2016; Georgiades et al., 2014; Verté et al., 2006), studies have also found subtypes distinguished by qualitative differences (Hu & Steinberg, 2009; Pichitpunpong et al., 2019). This is likely due to the sample size needed to identify “well-separated” classes with qualitative variations versus those with simpler models and fewer latent variables (Nylund-Gibson & Choi, 2018). Class 1 displayed a higher probability of maladaptive tendencies and maternal history of mental illness compared to the overall group. Our findings and existing literature suggest a positive correlation between behavioural co-morbidities including emotional dysregulation in the child and a maternal history of mental illness such as anxiety and depression. A latent class analysis by Wiggins et al. (2019) on 672 autistic preschool children identified a similar trend where maternal anxiety and depression were associated with 4.4 times higher odds of child phenotype with mild language and motor delay with dysregulation (Wiggins et al., 2019). Further, autistic individuals who exhibit more teacher-reported internalising features and parent-reported behavioural problems were more likely to have a history of maternal recurrent depression (Cohen & Tsiouris, 2006).

Class 2 was characterised by significant concerns in all behavioural domains associated with significant involvement of natal and postnatal determinants. Montogomery et al. (2023) performed a latent profile analysis of variables reflecting core autism traits, psychiatric and medical comorbidity on a sample of 754 children with autism and identified a subgroup of individuals with significant social communication and cognitive difficulties as well as increased sensory seeking behaviours (Montgomery et al., 2023). Further, a study by Momany et al. (2023) based on neonatal latent class analysis with variables including birthweight, gestational age, and the diagnostic status of common neonatal morbidities followed by analysis of covariance to examine eighteen-month neurodevelopmental scores by latent class identified 5 subgroups (Momany et al., 2023). They included complicated delivery, minor illness, and critically ill classes which attained lower neurodevelopmental scores compared to the healthy class, analogous to the pattern suggested by Class 2 (Momany et al., 2023). A notable difference in methodology is that Momany et al. (2023) used eighteen-month neurodevelopmental scores, whereas in the current study, age at the time of behavioural and sensory scoring was not accounted for. Existing literature emphasises the shared aetiologies of natal and postnatal determinants such as pregnancy and birth complications, neonatal physiological stress and inflammation, which can lead to future neurodevelopmental and behavioural concerns (Cheong et al., 2020; O’Shea et al., 2014).

Class 3 identified a subgroup with least concerns in all domains including sensory and behavioural adaptive skills relative to the overall group. Growth modelling studies indicate that core autism traits such as reduced adaptive functioning and communication difficulty in children improve over time and development (Baghdadli et al., 2012; Bal et al., 2015; Smith et al., 2012). Our findings suggest that Class 3, with the lowest probability of sensory and behavioural skills concerns, also had least probability of exposure to perinatal determinants. This highlights the cumulative impact of environmental and psychosocial determinants as ‘second hits’ may have an impact on severity of core-autism traits with those in Class 3 having the least exposure to perinatal issues and least concerns. This is consistent with available evidence about the greater likelihood of history of autoimmune diseases – rheumatoid arthritis, celiac disease, and type 1 diabetes – and serum anti-fetal brain antibodies etc. in mothers of autistic children (Atladóttir et al., 2009; Careaga et al., 2010).

Class 4 was characterised by the highest sensorily impairment in the overall group, associated with the highest exposure to perinatal determinants. A model-based cluster analysis conducted by Lane et al. (2014) on a smaller sample size of 228 autistic individuals examining autism symptom severity, sensory differences, and non-verbal intelligence quotient identified a distinct subgroup of individuals with generalised difficulty across all sensory domains, similar to Class 4 (Lane et al., 2014). However, Lane et al. (2014) identified subgroups of cases with similar sensory disabilities using cluster analysis, while our study used latent class analysis to find convergence based on item scores of variables. Previous studies have suggested the prevalence of sensory differences in autistic children to be as high as 92% and subgrouping based on sensory profile may have clinical implications in matching the right supports (Tomchek & Dunn, 2007). Class 4, much like the other subtypes, suggested a positive association between perinatal factors and clinical features of autism. Consistent with this finding, a study by Traver et al. (2021) found that autistic children with a maternal history of pregnancy-induced hypertension and pregnancy complications such as placental pathology presented with greater behavioural and communication disability (Traver et al., 2021).

The study design had both strengths and limitations. In terms of study strengths, the minimal set of inclusion criteria of the AAB allows for inclusion of children with a diverse range of clinical phenotypes and language and intellectual capacities including minimally verbal children, or children with a co-occurring cognitive impairment who are frequently excluded from projects involving biological sample collection. In addition, the large sample size of 1168, has provided adequate power to recognise well-distinguished groups. Latent class analyses with low sample sizes are subject to limitations such as failure to converge, poor functioning fit indices and failure to reveal classes with low memberships (Nylund-Gibson & Choi, 2018).

We also note several limitations. The variables used in the analysis do not include medical or psychiatric comorbidities of the child, which evidence shows can be significant contributors to an autistic child’s phenotype and family functioning, and thus may be crucial to identifying distinct subgroups (Hyman et al., 2020; Montgomery et al., 2021). The data collected within the AAB is also reliant on parent-reporting and therefore is prone to recall bias. Moreover, comparison with existing literature is challenging as to the authors’ knowledge, only a handful of studies have considered perinatal factors and investigated their association with behavioural and sensory manifestations. Direct comparison with existing subtyping studies is limited because of significant diversity in variables chosen for analyses.

## Conclusion

This study identified four subgroups within the AAB dataset that were distinguished based on behavioural skills and sensory impairments, and exposure to perinatal determinants. Our findings emphasise the relevance of perinatal determinants in distinct autism phenotypes. Further research is indicated for validation of the subgroups recognised. The identification of clinically meaningful subgroups and patterns between perinatal factors and core autism traits can pave the way for matching the right intervention and supports. Our findings also highlight a positive association between perinatal determinants and impairment in sensory processing and behavioural skills of autistic children. This association should be explored further in future research as replication of our findings is warranted to validate the subtypes identified. With distinct subgroups in place, practitioners will be able to classify children early in life based on perinatal determinants. This provides opportunities for early and individualised management of children’s behavioural and sensory profiles, which may improve the overall quality and wellbeing of both children and their families. Such individually tailored intervention and supports is expected to result in the best possible outcomes and developmental trajectories for children including better family functioning and societal participation.

## Data Availability

Data is available upon formal request to Autism CRC (autismcrc.com.au/biobank).
